# TIMP‐2 Modulates 5‐Fu Resistance in Colorectal Cancer Through Regulating JAK–STAT Signalling Pathway

**DOI:** 10.1111/jcmm.70470

**Published:** 2025-03-21

**Authors:** Chuchu Xu, Renjun Zhu, Qingfeng Dai, Yaoqing Li, Gengyuan Hu, Kelong Tao, Yuhong Xu, Guangen Xu, Guolin Zhang

**Affiliations:** ^1^ Department of Gastrointestinal Surgery Shaoxing People's Hospital Shaoxing Zhejiang Province China; ^2^ Department of Emergency Shaoxing People's Hospital Shaoxing Zhejiang Province China; ^3^ Zhijiang College, Zhejiang University of Technolog Shaoxing Zhejiang Province China; ^4^ Department of Gynaecology Shaoxing People's Hospital Shaoxing Zhejiang Province China

**Keywords:** 5‐Fu, colorectal cancer, drug resistance, JAK–STAT signalling pathway, TIMP‐2

## Abstract

The main reason for the failure of chemotherapy therapies based on 5‐Fluorouracil (5‐Fu) is the development of resistance to 5‐Fu in cancer patients, particularly those with colorectal cancer. Tissue inhibitor of metalloproteinases 2 (TIMP‐2) has been shown to be associated with colorectal cancer (CRC), but its correlation with 5‐Fu resistance in colorectal cancer has not been thoroughly studied. We screen the expression of different cytokines through Cytokine array. CCK‐8 assay was conducted to evaluate the IC_50_ of 5‐Fu and cell proliferation. ELISA and RT‐qPCR were performed to detect TIMP‐2 expression levels in cells and patient serum. Western blotting was utilised to analyse the differences in the expression of proteins related to signalling pathways in cells. Through cytokine array screening, we found that the expression of TIMP‐2 was significantly increased in CRC drug‐resistant cell lines. In addition, the expression of TIMP‐2 in the serum of patients with CRC resistance to 5‐Fu was significantly increased. Subsequent mechanistic experiments showed that TIMP‐2 regulated the resistance of CRC cells to 5‐Futhrough the JAK–STAT signalling pathway. Moreover, anti‐TIMP‐2 antibody or small molecule drug LY2784544 targeting the JAK–STAT signalling pathway can effectively reverse the resistance of CRC cells to 5‐Fu. It is exactly TIMP‐2 that mediates the resistance of CRC to 5‐Fu through the JAK–STAT signalling pathway. Targeting drugs for TIMP‐2 or the JAK–STAT signalling pathway are expected to be opportunities to reverse 5‐Fu resistance in CRC.

## 1 Introduction

Colorectal cancer (CRC) is the third most common cause of cancer mortality worldwide [1], of which 20% has metastasised at presentation [2]. Although synthetic treatments have demonstrated substantially increased cancer survival during the past 40 years, the 5‐year survival rate of CRC only increased from 51% to 65% [3]. The status of 5‐fluorouracil (5‐Fu) as a significant treatment for CRC is unshakable in neoadjuvant, adjuvant, or palliative therapies [4]. 5‐Fu, an antimetabolite drug, affects biosynthesis by inhibiting thymidylate synthase (TS) or introgressing its metabolites into RNA [5]. However, drug resistance emerges after several cycles of 5‐Fu‐based chemotherapy, which causes tumour progression and recurrence [6].

Different mechanisms reported can explain 5‐Fu resistance, such as cell cycle perturbation, tumour microenvironment (TME) regulation, miRNA dysregulation, DNA methylation, and demethylation [7, 8, 9, 10]. In previous studies, Lu et al. found that chemokine ligand 21 (CCL21) could trigger the PI3K/AKT signalling pathway in CRC cells to promote 5‐FU chemoresistance [11]. Dabkeviciene et al. reported that 5‐Fu could upregulate Interleukin‐1α (IL‐1α) and stimulate the CXCL8–CXCR1/2 pathway in 5‐Fu‐chemoresistant HCT116 cells, which contributed to tumour growth and invasion [12]. In conclusion, cytokines play a huge role in tumorigenesis, metastasis, as well as TME‐associated resistance [7, 13, 14, 15].

The matrix metalloproteinases (MMPs) are a family of endopeptidases present intracellularly, at membrane junctions that degrade the extracellular matrix [16]. Matrix metalloproteinase 2 (MMP‐2) is one of the MMPs family associated with tumour growth, invasion, metastasis, and neoangiogenesis [17, 18]. The function of MMP‐2 is inhibited reversibly by tissue inhibitor of metalloproteinase 2 (TIMP‐2), a natural inhibitor expressed constitutively at the surface of the cells and as soluble forms in most tissues [19, 20].

TIMP‐2 exerts a dual effect on tumour cells. Both over‐ and under‐expression of TIMP‐2 have been reported in different forms of cancer, including lung, breast, gastric, colorectal, and cervical cancers [21, 22, 23, 24, 25], which indicated quite the opposite prognosis [25, 26]. In CRC, Wang et al. found that low expression of TIMP‐2 was associated with poor prognosis, and lentivirus‐mediated overexpression or knockdown of TIMP‐2 could alter the invasion and migration of HCT 116 cells [27]. Furthermore, many studies have reported the relationship between TIMP‐2 and chemotherapy resistance of breast, ovarian cancers, and melanoma [28, 29, 30]. However, the evidence on TIMP‐2 in CRC cell resistance to 5‐FU was limited. It is noteworthy to figure out the molecular mechanism inherently and attempt to reverse chemoresistance.

Secreted proteins, especially cytokines, mainly rely on signalling pathways to regulate gene phenotypes [31]. The JAK–STAT signalling pathway is closely associated with immune fitness, inflammation, cell proliferation, differentiation, and apoptosis. It consists of three parts: tyrosine kinase‐related receptor to receive signals, tyrosine kinase JAK to transmit signals, and transcription factor STAT to produce effects, respectively [32, 33, 34]. Numerous cytokines and growth factors have showed to transmit signals through the JAK–STAT signalling pathway, including interleukin 2 (IL‐2), epidermal growth factor (EGF), platelet‐derived growth factor (PDGF), and interferon (IFN) [32, 35]. They unite with the binding site of tyrosine kinase JAK through the intracellular segment of the receptor on the cell membrane. After autophosphorylation, JAK transmits the signal forward and activates the STAT protein to modulate the transcriptional activity of the downstream targets [32, 33, 35].

STAT3 has been reported to be related to tumour cell growth, immunosuppression, and chronic inflammation [34]. Aberrant STAT3 expression via IL‐6 proliferation [36] and lncRNA Casc2 lost [37] have been shown to have carcinogenic effects on various tumours, including CRC [38, 39]. Meanwhile, the upregulation of STAT1 has been demonstrated to be associated with chemoresistance of doxorubicin, cisplatin and docetaxel in ovarian and prostate cancers [40, 41]. Some researchers have found the relationship between JAK2/STAT3 signalling and gemcitabine resistance in pancreatic and biliary tract cancer [42, 43]. However, the roles of this pathway in 5‐FU–emoresistant CRC and how to combat 5‐Fu resistance in clinical patients have been unknown.

In this study, we constructed 5‐Fu‐resistant CRC cell lines and found that TIMP‐2 was significantly increased in the resistant cell lines via cytokine microarray and transcriptome sequencing. Recombinant TIMP‐2 could induce sensitive CRC cell lines resistant to 5‐Fu, which could be reversed by TIMP‐2 antibody. Higher levels of p‐JAK2 and p‐STAT5 proteins were detected in 5‐Fu‐resistant cell lines. Further analysis showed that the JAK–STAT signalling pathway was involved in the 5‐Fu chemoresistance of CRC via TIMP‐2, which could be inhibited by the targeted small molecule drug LY2784544. Our findings suggested that TIMP‐2 might regulate the resistance of CRC to 5‐Fu through the JAK–STAT signalling pathway.

## 2 Materials and Methods

### 2.1 Antibodies and Reagents

The antibodies were obtained from the following companies: R&D systems (TIMP‐2 antibody), Cell Signalling Technology (GAPDH [Cat No. 97166]), Aladdin Technology (LY2784544 [Cat No. 1229236–86‐5]) and Hangzhou Fude Biological Technology (HRP‐conjugated antibodies). The recombinant TIMP‐2 was purchased from PeproTech. 5‐Fu was supplied by MedChemExpress.

### 2.2 Cell Culture

American Type Culture Collection (ATCC, Manassas) provided us with HCT116 cells and DLD‐1 cell lines for a fee. Dulbecco's Modified Eagle Medium (DMEM) with higher glucose levels (Genom) was the medium we used to cultivate the HCT116 cell line. RPMI‐1640 (Genom) was the medium we used to cultivate the DLD‐1 cell line. Both of the above media were supplemented with 10% fetal bovine serum (GIBCO). The incubator is kept at 37°C and 5% CO_2_ to fit the growth of cells.

Cultivation of 5‐Fu‐resistant CRC cell lines: Two CRC cell lines were alternately cultured in a medium with increasing concentrations of 5‐Fu and a medium without 5‐Fu, and their IC_50_ values were checked regularly. When the two cell lines can grow smoothly in a constant concentration of 5‐Fu medium, it proves that the drug‐resistant cell line has been successfully constructed.

### 2.3 Cell Viability Assay

The Cell‐Counting Kit‐8 (CCK8 kit) provided by Dojindo Molecular Technologies was used to detect cell viability and calculate IC_50_. Each experiment was repeated more than three times and was carried out in strict accordance with the instructions.

### 2.4 Cytokine Array

The Array Map for Human Cytokine antibody Array (ab193656) from Abcam was used to detect cytokines in cell culture medium. 24 h before the screening, DLD‐1 5‐FuS and DLD‐1 5‐FuR cells were cultured in RPMI‐1640 medium without fetal bovine serum. The cytokine detection process was standardised in accordance with the manufacturer's protocol, including internal control settings and intragroup controls, etc.

### 2.5 Enzyme‐Linked Immunosorbent Assay (ELISA)

ELISA kit (Elabscience) for TIMP‐2 was specially used to detect the content of TIMP‐2 in cell culture medium. The operation process was carried out in accordance with the instructions. Triplicate wells were carried out in 3 independent experiments.

### 2.6 RNA Isolation and RTq‐PCR

We used the Trizol reagent (Invitrogen) to extract the total RNA. The cDNA reverse transcriptase kit (Takara) synthesised cDNA. The LightCycler 480 real‐time PCR system (Roche, Mannheim) undertook the SYBR Green‐based (Takara) quantitative real‐time PCR (RTq‐PCR) process. Glyceraldehyde‐3‐phosphate dehydrogenase (GAPDH) was the choice of internal control. The 2^−ΔΔCq^ relative quantification method was used to calculate the expression level of target mRNA.

### 2.7 Patients

We collected serum samples of CRC patients from Sir Run Run Shaw Hospital, School of Medicine, Zhejiang University, and Shaoxing People's Hospital (Shaoxing Hospital, Zhejiang University School of Medicine) from 2010 to 2018. A total of 40 patients were included in the study and divided into two groups based on whether they were resistant to 5‐Fu during chemotherapy. This classification was determined by evaluating tumour regression within a 6‐month period after the administration of 5‐Fu. In the course of chemotherapy involving 5‐Fu‐based chemotherapeutic agents, if a patient's tumour does not show signs of progression during this time, we categorise these patients as being sensitive to 5‐Fu(5‐Fu sen). Conversely, if there is evidence of tumour progression, the patient is deemed to be resistant to 5‐Fu(5‐Fu res). To mitigate the impact of cytokine degradation due to long‐term storage, patient serum was aliquoted and stored at −80°C immediately after collection. This approach minimises freeze–thaw cycles and maintains cytokine stability. Additionally, serum processing was conducted promptly post‐collection to prevent cytokine release from blood cells. The tumour types of CRC patients are checked by two or more senior pathologists.

### 2.8 WB Analysis

Cells were lysed by RIPA lysis buffer (Solarbio Life Sciences). Bicinchoninic acid assay (BCA, Beyotime Institute of Biotechnology) was used to determine protein concentrations. We used 10% SDS‐PAGE (Beyotime Institute of Biotechnology) to separate the protein and then used polyvinylidene fluoride membranes (Immobilon‐P) for protein transfer. Membranes were soaked in 5% dried skimmed milk at room temperature for 1 h and then incubated overnight in the presence of primary antibodies at 4°C. IgG conjugated goat anti‐rabbit was used as secondary antibodies for incubation, and the incubation conditions were 1–2 h at room temperature. The strips were exposed after treatment with enhanced chemiluminescence detection reagent (Hangzhou Fude Biological Technology).

### 2.9 Statistical Analysis

SPSS (version 22.0), Graph Pad Prism (version8.0) and ImageJ (version 2.0) were used to analyse data from three independent experiments. We presented the data in the form of means ± SD. The one‐sample Kolmogorov–Smirnov test was used to test whether the experimental data conforms to the normal distribution. When analysing the two groups' experimental results, we used independent sample t‐test or non‐parametric test. For the analysis between different groups, we adopted one‐way ANOVA multiple comparison analysis with Tukey's posttest or two‐way ANOVA with Fisher's LSD test. *p* ≤ 0.05 was considered significant and *p* values were two‐sided in all cases.

### 2.10 Ethical Considerations

The clinical patient part of this experiment was approved by the ethical approval agency at Shaoxing People's Hospital (Shaoxing Hospital, Zhejiang University School of Medicine), and the approved number was 2021‐K‐Y‐158‐01. All experimental procedures were subject to the supervision and review of the ethics committee.

## 3 Results

### 3.1 Identification of TIMP‐2 as a Key Factor That Regulates CRC Resistance to 5‐Fu

**FIGURE 1:**
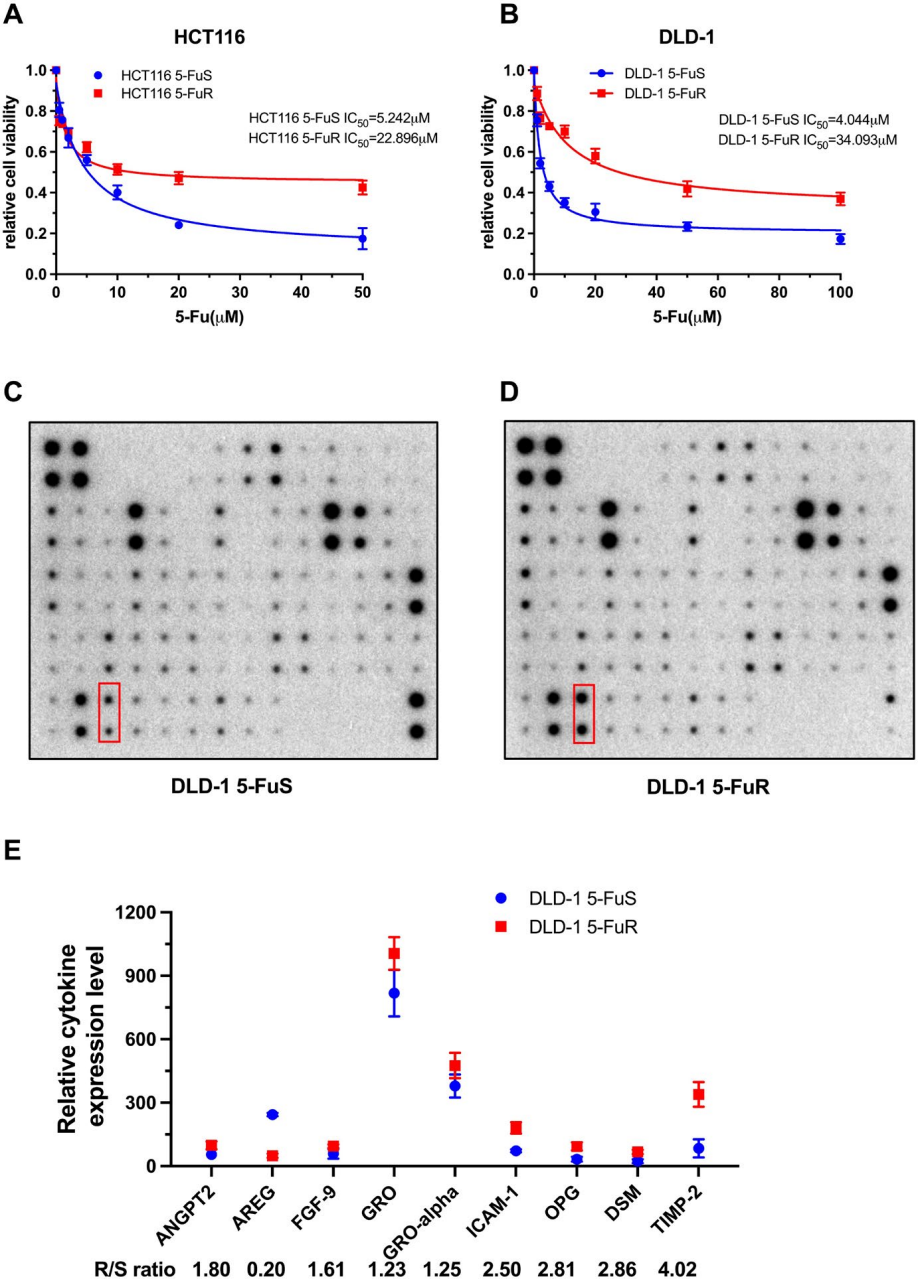
Identification of TIMP‐2 that promotes 5‐Fu‐resistant colorectal cancer cells resistant to 5‐Fu.(A, B) Viability of HCT116 5‐FuS and HCT116 5‐FuR cells, DLD‐1 5‐FuS and DLD‐1 5‐FuR cells treated with different concentrations of 5‐Fu for 3 days. The IC_50_ was indicated.(C, D) Dot plot of relative cytokine expression levels using a cytokine array (Abcam‐ab193656) in the cell culture medium of DLD‐1 5‐FuS and DLD‐1 5‐FuR cells. The red frame enclosed the spots of TIMP‐2. (E) Semi‐quantitative analysis of cytokines with large differences in the cell culture medium of DLD‐1 5‐FuS and DLD‐1 5‐FuR cells.

First, we established 5‐Fu resistant CRC cells using procedures as described in previous methods. Those cell lines that can tolerate 5‐Fu stimulation were named HCT116 5‐FuR cells and DLD‐1 5‐FuR cells, while those original cell lines were named HCT116 5‐FuS cells and DLD‐1 5‐FuS cells. After 8 months of the 5‐Fu resistance culture process, the IC_50_ (the 50% inhibitory concentration) of the drug‐resistant cell lines showed a huge difference compared with that of the non‐drug‐resistant cell lines. Specifically, the IC_50_ of HCT116 5‐FuR cells was 4.368 times that of the original cell line (Figure 1A), and the IC_50_ of DLD‐1 5‐FuR cells was 8.431 times that of the original cell line (Figure 1B). This confirmed that we had successfully constructed two 5‐Fu‐resistant CRC cell lines.

To identify the cytokines responsible for CRC resistance to 5‐Fu, we screened the culture medium of DLD‐1 5‐FuS and DLD‐1 5‐FuR cells using a cytokine array (The Array Map for Human Cytokine antibody Array [ab193656]). The specific spot difference was presented in Figure 1C,D. The red frame enclosed the spots of TIMP‐2. Through semi‐quantitative analysis of spots, we found cytokines with large differences in expression, including OPG, DSM, TIMP‐2, etc. Among them, TIMP‐2 expressed the biggest difference in the culture medium of the two cell lines, with a difference of 4.02 times (Figure 1E).

**FIGURE 2:**
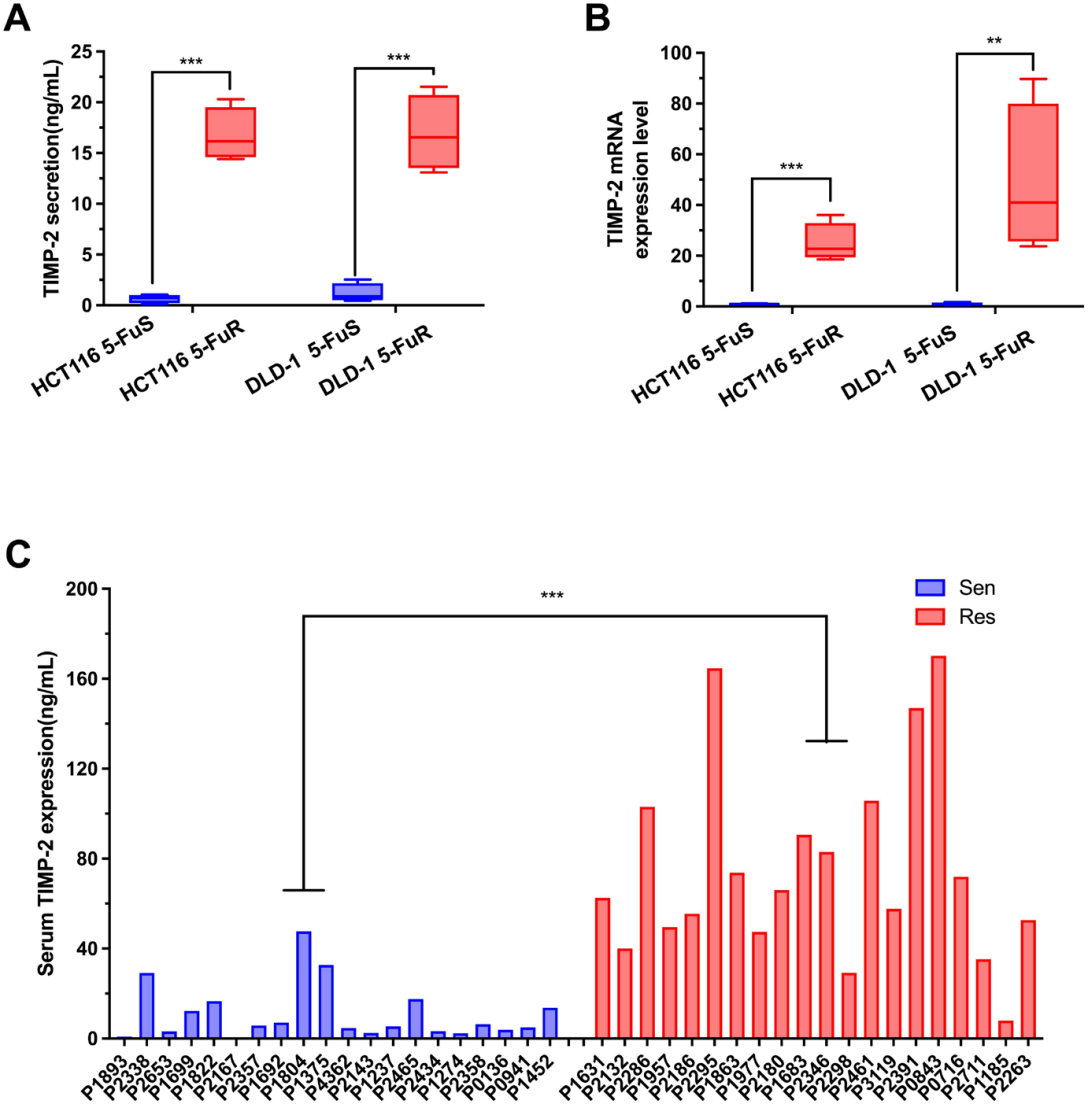
TIMP‐2 is elevated in 5‐Fu resistant colorectal cancer cells and patients. (A) Difference in TIMP‐2 protein expression level in the paired HCT116 5‐FuS and HCT116 5‐FuR cells, DLD‐1 5‐FuS and DLD‐1 5‐FuR cells. (B) Difference in TIMP‐2 mRNA expression level in the paired HCT116 5‐FuS and HCT116 5‐FuR cells, DLD‐1 5‐FuS and DLD‐1 5‐FuR cells. (C) Comparison of the levels of TIMP‐2 protein measured by ELISA in 5‐Fu sensitive (*n* = 20) and resistant (*n* = 20) colorectal cancer patients, patients' information is shown in Table 1. Sen, sensitive cases. Res, resistant cases. **p < 0.01, ***p < 0.001 by unpaired Student's *t*‐test. Data from triplicate wells in 3 independent experiments.

Furthermore, we used ELISA and real‐time quantitative PCR to carefully detect the expression of TIMP‐2. Regardless of the transcriptome or protein expression level, HCT116 5‐FuR cells and DLD‐1 5‐FuR cells showed higher levels of TIMP‐2 than non‐resistant cell lines (Figure 2A,B). In addition, we also carried out relevant verifications on clinical patients. We clinically screened 20 patients with CRC who were resistant to 5‐Fu and 20 patients who were not resistant to 5‐Fu. The patients' specific information and clinical characteristics were shown in Tables 1 and 2. It was obvious that the expression level of TIMP‐2 in the serum of 5‐Fu‐resistant CRC patients was significantly higher than that of non‐resistant (Figure 2C).

**TABLE 1:** Detailed clinical characteristics of patients.

	Patient	Age (years)	Sex	Stage	Histology	Chemotherapy
5‐Fu sen	P0136	48	F	IVA	Adenocarcinoma	5‐Fu+Oxaliplatin
	P0941	66	M	IVB	Adenocarcinoma	5‐Fu+Irinotecan+Oxaliplatin+Bevacizumab
	P1237	68	F	IIIC	Mucus adenocarcinoma	5‐Fu+Irinotecan+Oxaliplatin
	P1274	56	M	IVA	Adenocarcinoma	5‐Fu+Oxaliplatin
	P1375	67	M	IVA	Adenocarcinoma	5‐Fu+Irinotecan+Oxaliplatin+Bevacizumab
	P1452	63	F	IVB	Adenocarcinoma	5‐Fu+Irinotecan+Oxaliplatin
	P1692	61	F	IVA	Adenocarcinoma	5‐Fu+Irinotecan+Oxaliplatin+Bevacizumab
	P1699	78	F	IVA	Mucus adenocarcinoma	5‐Fu+Oxaliplatin+Bevacizumab
	P1804	21	F	IIIC	Mucus adenocarcinoma	5‐Fu+Irinotecan+Oxaliplatin
	P1822	76	M	IVB	Adenocarcinoma	5‐Fu+Irinotecan+Oxaliplatin+Bevacizumab
	P1893	55	M	IVA	Adenocarcinoma	5‐Fu+Oxaliplatin
	P2143	57	M	IVB	Adenocarcinoma	5‐Fu+Oxaliplatin+Bevacizumab
	P2167	66	M	IVA	Adenocarcinoma	5‐Fu+Irinotecan+Oxaliplatin+Bevacizumab
	P2338	66	M	IVB	Adenocarcinoma	5‐Fu+Irinotecan+Oxaliplatin+Cetuximab
	P2357	70	M	IIIB	Adenocarcinoma	5‐Fu+Irinotecan+Bevacizumab
	P2358	77	M	IVB	Adenocarcinoma	5‐Fu+Irinotecan+Oxaliplatin
	P2434	76	M	IIIC	Mucus adenocarcinoma	5‐Fu+Irinotecan+Oxaliplatin +Bevacizumab
	P2465	61	F	IVB	Adenocarcinoma	5‐Fu+Irinotecan+Oxaliplatin+Bevacizumab
	P2653	65	M	IIIC	Adenocarcinoma	5‐Fu+Irinotecan+Oxaliplatin+Cetuximab
	P4362	72	F	IVA	Adenocarcinoma	5‐Fu+Irinotecan+Oxaliplatin+Cetuximab
5‐Fu res	P0716	78	M	IVA	Adenocarcinoma	5‐Fu+Irinotecan+Bevacizumab
	P0843	52	F	IVB	Adenocarcinoma	5‐Fu+Irinotecan+Bevacizumab
	P1185	80	M	IVB	Adenocarcinoma	5‐Fu+Irinotecan+Bevacizumab
	P1631	66	M	IVA	Adenocarcinoma	5‐Fu+Irinotecan+Oxaliplatin +Bevacizumab
	P1683	35	F	IVB	Adenocarcinoma	5‐Fu+Oxaliplatin
	P1863	47	F	IVB	Mucus adenocarcinoma	5‐Fu+Irinotecan+Oxaliplatin +Bevacizumab
	P1957	55	M	IVB	Adenocarcinoma	5‐Fu+Irinotecan+Oxaliplatin+Cetuximab
	P1977	51	F	IVB	Adenocarcinoma	5‐Fu+Irinotecan+Oxaliplatin+Bevacizumab
	P2132	55	M	IVB	Adenocarcinoma	5‐Fu+Irinotecan+Oxaliplatin+Bevacizumab
	P2180	53	F	IVB	Adenocarcinoma	5‐Fu+Oxaliplatin
	P2186	65	M	IIIC	Adenocarcinoma	5‐Fu+Irinotecan+Oxaliplatin
	P2263	63	F	IIIC	Adenocarcinoma	5‐Fu+Irinotecan+Oxaliplatin +Cetuximab
	P2286	59	M	IVA	Adenocarcinoma	5‐Fu+Irinotecan+Oxaliplatin+Cetuximab+Bevacizumab
	P2295	48	M	IIIC	Adenocarcinoma	5‐Fu+Irinotecan+Oxaliplatin+Bevacizumab
	P2298	41	F	IVA	Adenocarcinoma	5‐Fu+Irinotecan+Oxaliplatin+Bevacizumab
	P2346	68	M	IIIC	Adenocarcinoma	5‐Fu+Irinotecan+Oxaliplatin
	P2391	69	F	IVA	Mucusadenocarcinoma	5‐Fu+Oxaliplatin

	P2461	71	M	IVA	Adenocarcinoma	5‐Fu+Irinotecan+Bevacizumab
	P2711	45	F	IVA	Mucus adenocarcinoma	5‐Fu+Oxaliplatin
P3119	56	M	IVB	Adenocarcinoma	5‐Fu+Irinotecan+Oxaliplatin+Cetuximab

**TABLE 2:** Description of clinical characteristics of patients.

Characteristics	Total	5‐Fu sen	5‐Fu res	OR	*p*
All Cases	40	20 (50.0%)	20 (50.0%)		
Age (years)					
≥ 65	19	12 (63.2%)	7 (36.8%)		
< 65	21	8 (38.1%)	13 (61.9%)	0.359	0.205
Gender					
Male	23	12 (52.2%)	11 (47.8%)		
Female	17	8 (47.1%)	9 (52.9%)	0.815	0.646
Stage					
IIIB	1	1 (100.0%)			
IIIC	8	4 (50.0%)	4 (50.0%)		
IVA	15	8 (53.3%)	7 (46.7%)		
IVB	16	7 (43.8%)	9 (56.2%)		0.240
Histological type					
Adenocarcinoma	33	16 (48.5%)	17 (51.5%)		
Mucus adenocarcinoma	7	4 (57.1%)	3 (42.9%)	0.706	1.000

*Note:* *p*‐value calculated by chi‐square test.

### 3.2 Alteration of TIMP‐2 Expression Correlated With 5‐Fu Resistance in CRC Cells

**FIGURE 3:**
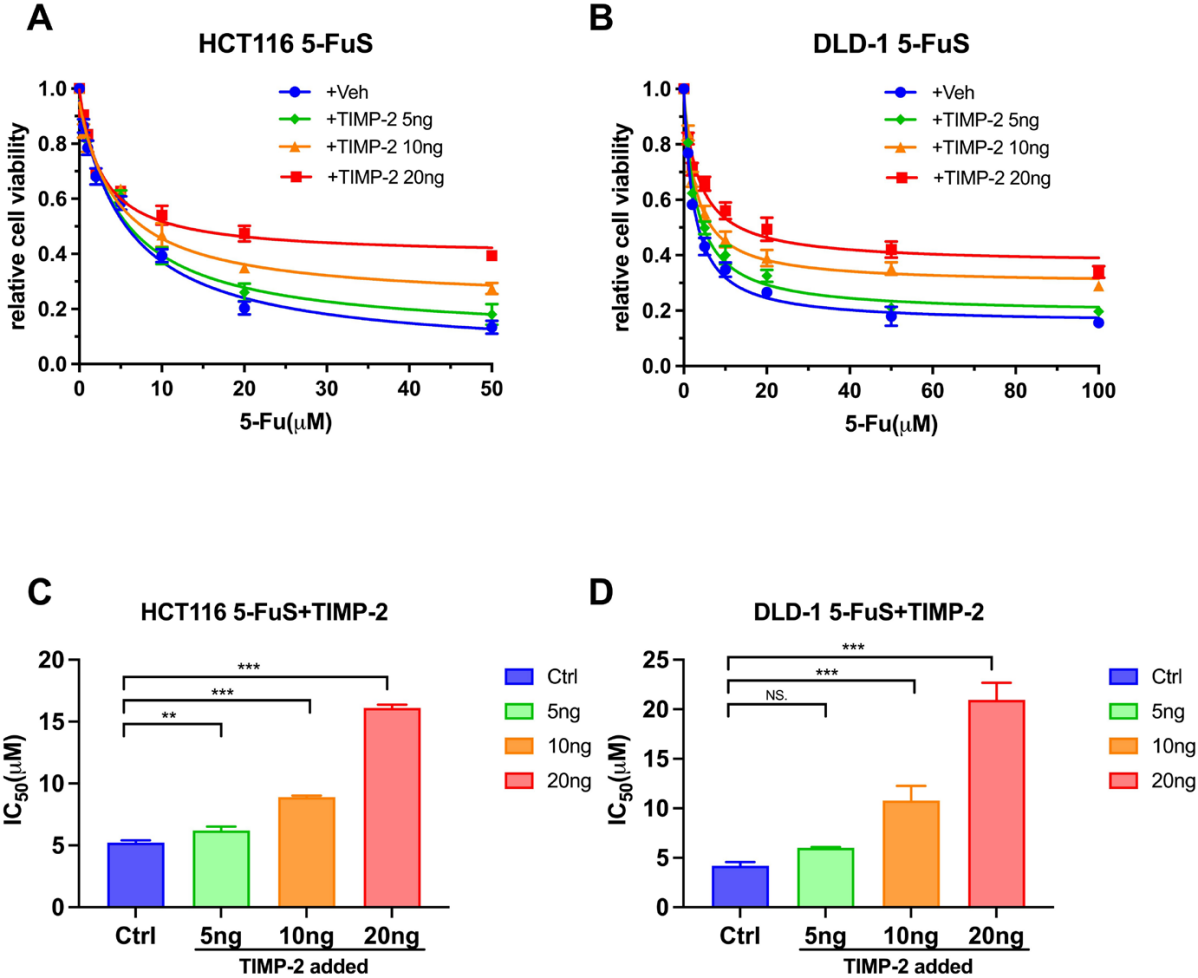
Up‐regulation of TIMP‐2 promotes 5‐Fu resistance of colorectal cancer cells. (A, B) Viability of HCT116 5‐FuS and DLD‐1 5‐FuS cells treated with 5‐Fu of increasing concentration for 3 days after co‐culturing with 5 ng/mL or 10 ng/mL or 20 ng/mL of recombinant TIMP‐2 for 6 h. (C, D) The 5‐Fu concentration of 50% inhibition of cell growth (IC_50_) of four groups of cells in A and B above. * **p < 0.01, ***p < 0.001 by unpaired Student's t‐test. Data from triplicate wells in 3 independent experiments.

We had found the differences in the expression of TIMP‐2 in drug‐resistant and non‐drug‐resistant cell lines, as well as clinical patients. We next considered whether TIMP‐2 was the most important cytokine that caused this change. In order to verify our conjecture, we added different concentrations of recombinant TIMP‐2 to the culture medium of HCT116 5‐FuS and DLD‐1 5‐FuS cells. Consistent with our hypothesis, non‐resistant cell lines showed resistance to 5‐Fu after adding recombinant TIMP‐2. In addition, as the concentration of the added recombinant TIMP‐2 was increased from 5 ng/mL to 20 ng/mL, the 5‐Fu resistance of TIMP‐2 to CRC non‐resistant cell lines became more obvious (Figure 3A,B). From the point of view of IC_50_, the IC_50_ of CRC cell lines with increasing concentrations of recombinant TIMP‐2 added was significantly higher than that of cell lines without addition (Figure 3C,D).

**FIGURE 4:**
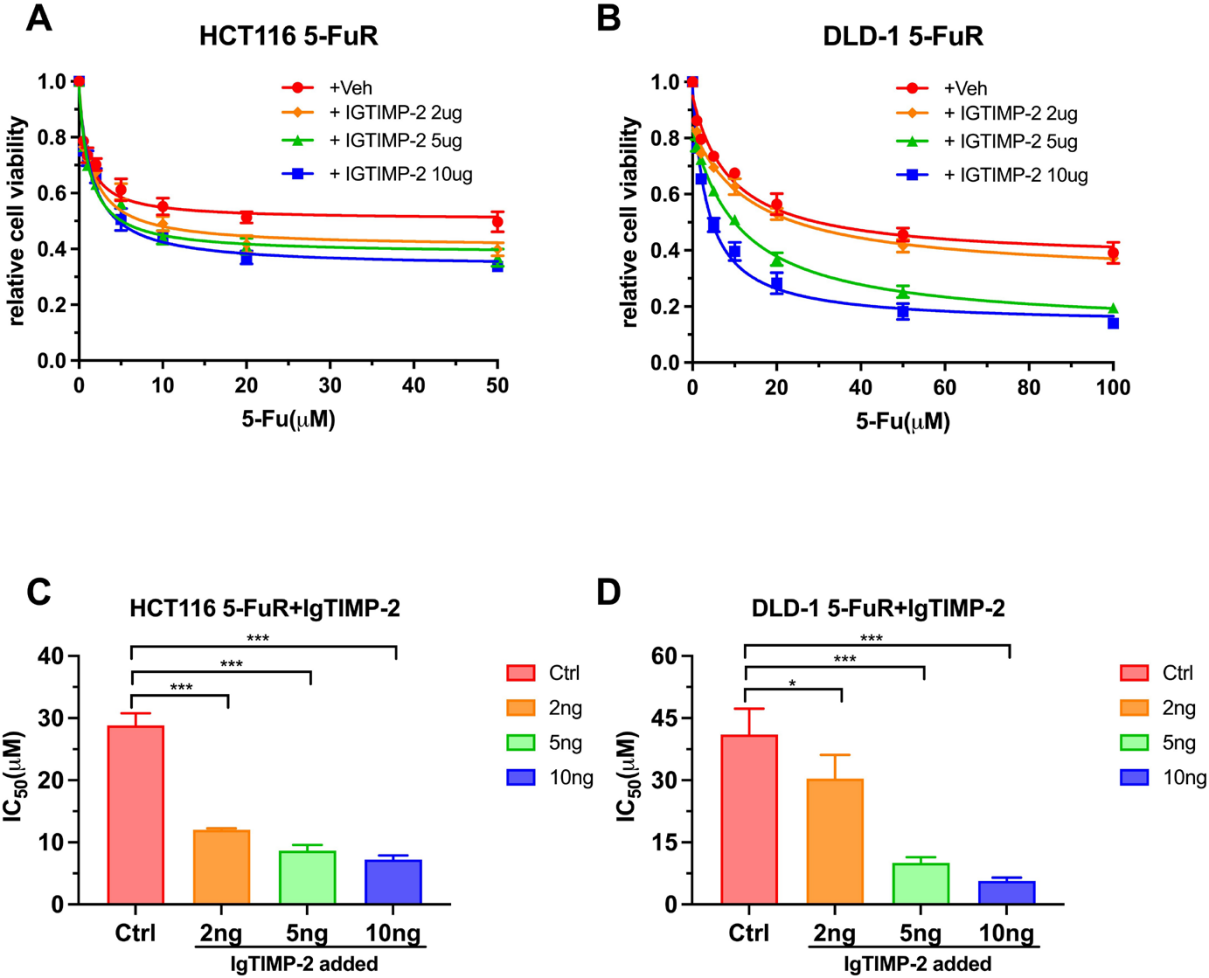
Neutralising TIMP‐2 reverses 5‐Fu resistance of colorectal cancer cells. (A, B) Viability of HCT116 5‐FuR and DLD‐1 5‐FuR cells treated with 5‐Fu of increasing concentration for 3 days after co‐culturing with a control IgG or a TIMP‐2 neutralising antibody (2 μg/mL or 5 μg/mL or 10 μg/mL) for 6 h. (C, D) The 5‐Fu concentration of 50% inhibition of cell growth (IC_50_) of four groups of cells in A and B above. *p < 0.05, **p < 0.01, ***p < 0.001 by unpaired Student's t‐test. Data from triplicate wells in 3 independent experiments.

Correspondingly, we added neutralising TIMP‐2 antibodies to the culture medium of HCT116 5‐FuR and DLD‐1 5‐FuR cells to observe whether TIMP‐2 was the most important cause of 5‐Fu resistance in CRC drug‐resistant cell lines. It was obvious that when a higher concentration of neutralising TIMP‐2 antibody was added to the culture medium of the drug‐resistant cell lines, the drug‐resistant cell lines became more resistant to 5‐Fu (Figure 4A,B). The IC_50_ of both HCT116 5‐FuR and DLD‐1 5‐FuR cells were significantly decreased, indicative of increased sensitivity of cells to 5‐Fu (Figure 4C,D). These experimental results showed that it was TIMP‐2, rather than other cytokines, that mainly caused the resistance of CRC cell lines to 5‐Fu.

### 3.3 TIMP‐2 Promotes 5‐Fu Resistance via Regulating JAK–STAT Activation in CRC

Now that it is known that TIMP‐2 is a key cytokine that causes CRC to be resistant to 5‐Fu, it becomes an important question which signalling pathway through which TIMP‐2 exerts the physiological function of cells. Previous articles reported that TIMP‐2 could co‐immunoprecipitate with JAK–STAT [44]. Therefore, we detected the key proteins of the JAK–STAT signalling pathway in two CRC cell lines, respectively.

**FIGURE 5:**
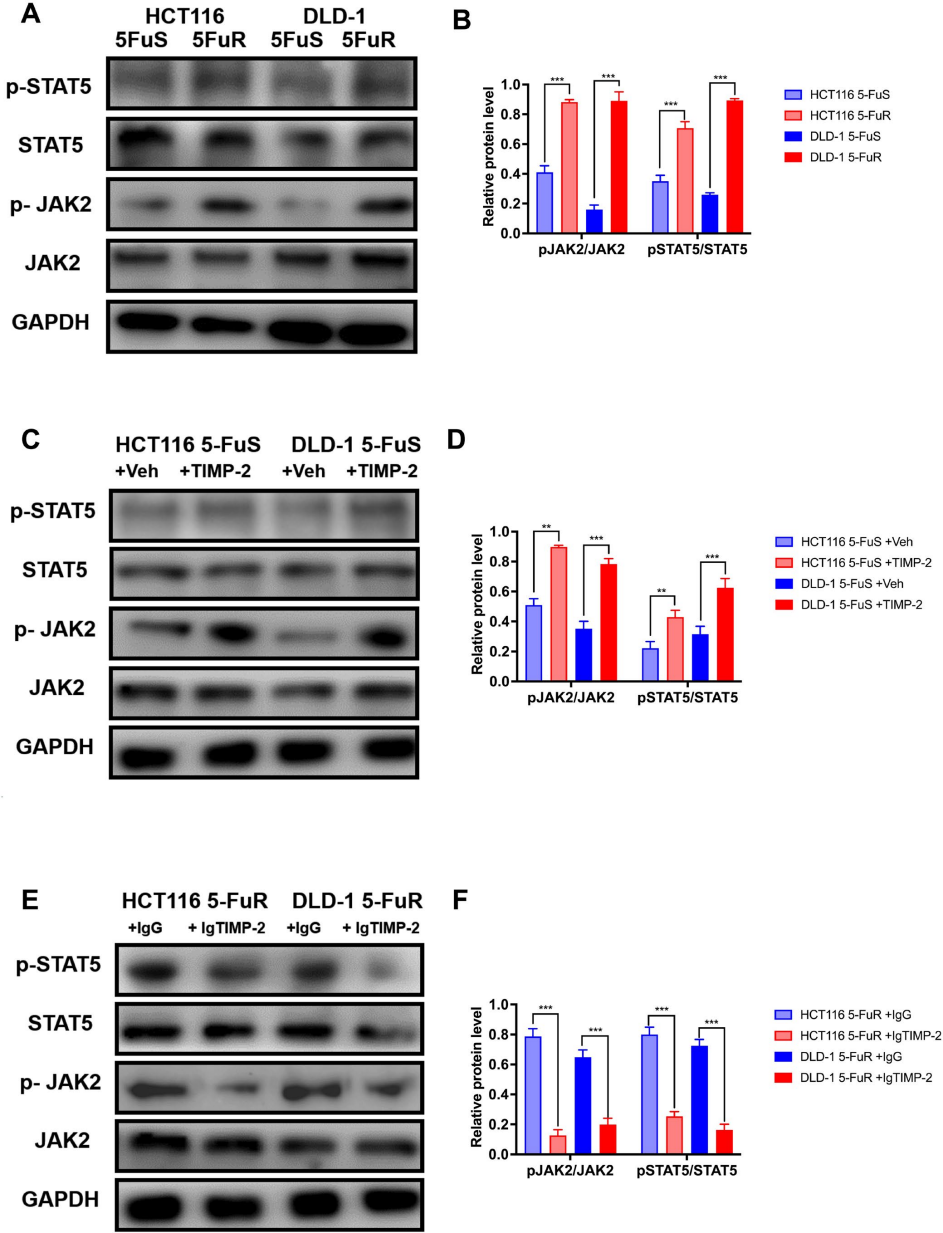
TIMP‐2 promotes 5‐Fu resistance via regulating JAK–STAT activation in colorectal cancer. (A) Immunoblotting of phosphorylation STAT5 and phosphorylation JAK2 in HCT116 5‐FuS cells and HCT116 5‐FuR, DLD‐1 5‐FuS cells and DLD‐1 5‐FuR cells. (B) Immunoblotting of phosphorylation STAT5 and phosphorylation JAK2 in HCT116 5‐FuS cells and DLD‐1 5‐FuS cells cultured with 10 ng/mL of recombinant TIMP‐2 for 6 h. (C) Immunoblotting of phosphorylation STAT5 and phosphorylation JAK2 in HCT116 5‐FuR cells and DLD‐1 5‐FuR cells cultured with control IgG or 5 μg/mL of TIMP‐2 neutralising antibody for 6 h. **p < 0.01, ***p < 0.001 by unpaired Student's t‐test. Data from triplicate wells in 3 independent experiments.

First, we examined the basic expression of the JAK–STAT signalling pathway in drug‐resistant and non‐resistant cell lines. HCT116 5‐FuR and DLD‐1 5‐FuR cells exhibited higher levels of phosphorylation of STAT5 and phosphorylation JAK2 than HCT116 5‐FuS and DLD‐1 5‐FuS cells (Figure 5A,B). When we added high concentrations of recombinant TIMP‐2 to the supernatant of non‐resistant cell lines to mimic TIMP‐2 overexpression, the expression of phosphorylation of STAT5 and phosphorylation of JAK2 was significantly increased (Figure 5C,D). Likewise, treatment with the TIMP‐2 neutralisation antibody resulted in a dramatically decreased level of phosphorylation of STAT5 and phosphorylation of JAK2 in both HCT116 5‐FuR and DLD‐1 5‐FuR cells (Figure 5E,F). In this way, our experiments basically proved that TIMP‐2 mediates the resistance of CRC to 5‐Fu through the JAK–STAT signalling pathway.

### 3.4 LY2784544 Targets JAK–STAT Signalling Regulating 5‐Fu Resistance in CRC

**FIGURE 6:**
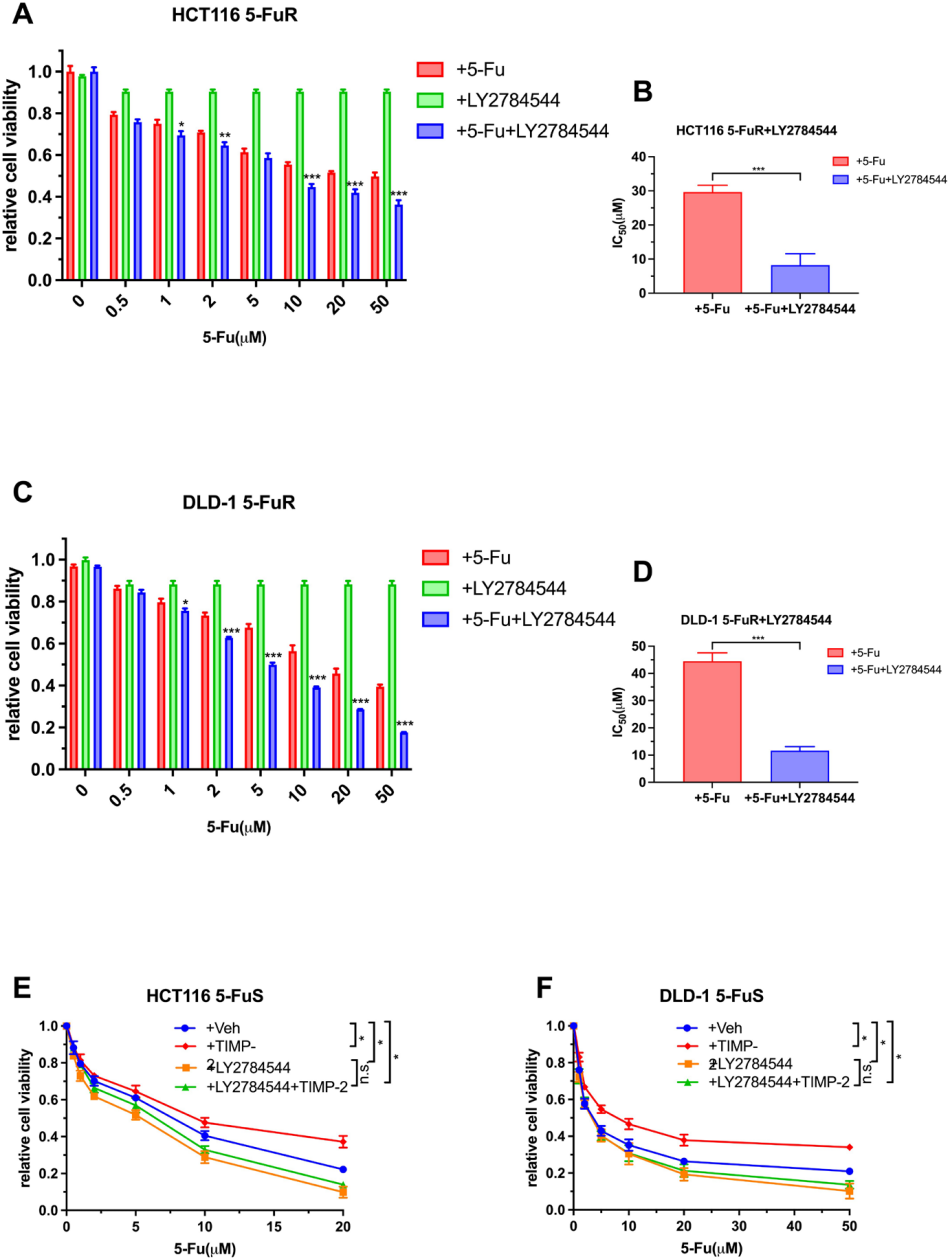
LY2784544 targets JAK–STAT signalling regulating 5‐Fu resistance in colorectal cancer. (A) The synergistic effects of LY2784544 and 5‐Fu on HCT116 5‐FuR cells. (B) The 5‐Fu concentration of 50% inhibition of cell growth (IC_50_) of two groups of cells in A above. (C) The synergistic effects of LY2784544 and 5‐Fu on DLD‐1 5‐FuR cells. (D) The 5‐Fu concentration of 50% inhibition of cell growth (IC_50_) of two groups of cells in C above. (E, F) Knockdown JAK–STAT by LY2784544 blocks TIMP‐2 induced 5‐Fu resistance in colorectal cancer cells. HCT116 5‐FuS and DLD‐1 5‐FuS cells were cultured with 0.1 μM of LY2784544 for 24 h and then cultured with recombinant TIMP‐2 (10 ng/mL) for 6 h, followed by increasing concentrations of 5‐Fu treatment for 3 days. (A–D) *p < 0.05, ***p < 0.001 by unpaired Student's t‐test. (E, F) *p < 0.05, **p < 0.01, ***p < 0.001 by one‐way ANOVA or two‐way ANOVA. Data from triplicate wells in 3 independent experiments.

Previous experiments confirmed to us that JAK–STAT signalling played a huge role in TIMP‐2‐mediated CRC resistance to 5‐Fu. From the perspective of clinical treatment, we try to verify whether LY2784544 can inhibit the resistance of CRC to 5‐Fu. We added different concentrations of 5‐Fu separately or LY2784544 at the same time to the culture medium of HCT116 5‐FuR cells to observe the efficacy of LY2784544 in reversing 5‐Fu resistance. What is more, we separately added LY2784544 to the culture medium of HCT116 5‐FuR cells to exclude its effect on cell viability. Consistent with our assumptions, LY2784544 and 5‐Fu had a strong synergistic effect on inhibiting the cell viability of HCT116 5‐FuR cells. In other words, LY2784544 showed a strong reversal effect against 5‐Fu resistance in CRC cell lines (Figure 6A). The difference in IC_50_ between the two groups was shown in Figure 6B. We conducted the same experiment in DLD‐1 5‐FuR cells and reached a consistent conclusion (Figure 6C,D).

In order to further clarify whether LY2784544 can reverse the 5‐Fu resistance of CRC cell lines caused by the cytokine TIMP‐2, we added recombinant TIMP‐2 and LY2784544 alone or in combination to the culture medium of the non‐5‐Fu‐resistant CRC cell lines. Recombinant TIMP‐2 can significantly increase the resistance of HCT116 5‐FuS and DLD‐1 5‐FuS cell lines to 5‐Fu, but its effect is significantly offset in the presence of LY2784544 (Figure 6E,F). When the JAK–STAT signalling pathway was suppressed, TIMP‐2‐induced 5‐Fu resistance was significantly inhibited. This provided strong evidence that TIMP‐2 regulated CRC resistance to 5‐Fu through JAK–STAT signalling and LY2784544 can reverse this process.

## 4 Discussion

5‐Fu is a drug widely used in tumour chemotherapy. It affects apoptosis, cell cycle changes, glucose metabolism, autophagy, and oxidative stress by affecting DNA synthesis. As 5‐Fu plays a fundamental role in the chemotherapy of digestive tract tumours, breast cancer, cervical cancer, and bladder cancer, it is still the key treatment drug for these tumours so far. However, it is precisely because of the wide application of 5‐Fu that various tumours gradually appear resistance to it in clinical treatment.

The issue of drug resistance in cancer patients is a global challenge. Although previous studies on the tumour microenvironment, LncRNA and Epithelial‐Mesenchymal Transition (EMT) have confirmed their relationship with tumour drug resistance, the specific pathophysiological mechanism behind it has not been fully elucidated [45, 46]. Specifically, in the study of 5‐Fu drug resistance in CRC, tumour microenvironment function, miRNA dysregulation, LncRNA changes, and epigenetic changes are potential research directions [7]. These intracellular and extracellular factors are inseparable from the important transition of cytokines.

Thus, after culturing and screening 5‐Fu‐resistant CRC cell lines, we screened a series of differentiated cytokines using cytokine array (The Array Map for Human Cytokine antibody Array (ab193656)). Double validation by semi‐quantitative analysis and ELISA confirmed that the expression of TIMP‐2 was significantly different between drug‐resistant cell lines and non‐drug‐resistant cell lines. TIMP‐2, known as tissue inhibitor of metalloproteinase 2, is differentially expressed in many tumours, including lung, breast, gastric, colorectal and cervical cancers [21, 22, 23, 24, 25], which also indicates that it may also have different expression levels in tumour patients. For a long time, we collected serum from CRC patients during chemotherapy and found that the expression level of TIMP‐2 in the serum of 5‐Fu‐resistant patients was much higher than that of non‐resistant patients (Figure 2C). It should be noted that the experimental results on patient serum have also been presented in papers published by our laboratory [47]. TIMP‐2 could also be used as an indicator to monitor the drug resistance status of CRC patients treated with 5‐Fu‐based chemotherapy. This indicates that TIMP‐2 indeed plays a pivotal role in the process of CRC resistance to 5‐Fu.

Due to experimental needs, this research project collected serum samples from CRC patients over many years. The untimely centrifugation and repeated freezing and thawing of serum can easily lead to a rapid decrease in the level of cytokines in serum, thus affecting the experimental results related to various cytokines. Therefore, we collected the patients' blood samples and sent them to the central laboratory for centrifugation within 30 min, and the serum obtained after centrifugation was divided into multiple EP tubes in small quantities for subsequent experiments. The aliquoted serum was stored in a −80°C freezer according to the label. After each experiment, a small amount of EP tube was taken out for the experiment, and the remaining serum was not put back in the refrigerator but was directly discarded. As far as possible, a small amount of cytokine levels will decrease during the freezing and thawing process. We adopt the method of centralised detection in the same batch at the same time to ensure that the cytokine levels are close to the real situation in the patient as much as possible.

In order to verify that it is TIMP‐2 rather than other cytokines that mainly caused the resistance of CRC cells to 5‐Fu, we added recombinant TIMP‐2 to the culture medium of non‐resistant cell lines and added neutralising TIMP‐2 antibodies to the culture medium of drug‐resistant cell lines respectively. The experimental results show that TIMP‐2 is the key factor for 5‐Fu to induce drug resistance in CRC cells, and this connection is significantly correlated with the concentration of TIMP‐2 in the culture medium. Now that this is clear from the perspective of cell experiments, we found the differential expression of the JAK–STAT signalling pathway in the two cell lines by screening signalling pathways. Similarly, we observed the same changes in the JAK–STAT cell signalling pathways after adding recombinant TIMP‐2 or neutralising TIMP‐2 antibodies to the culture medium. Consistent with our guess, TIMP‐2 indeed mediated the resistance of CRC cells to 5‐Fu through the JAK–STAT signalling pathway.

Our laboratory results not only clarify the role of TIMP‐2 in drug resistance, but also suggest that JAK–STAT is a downstream pathway of TIMP‐2. However, this project has not further clarified the TIMP‐2‐related gene status, RNA coding, and other related aspects. The clarification of these details will help us to further explore the cytological mechanism of CRC resistance to 5‐Fu. In addition, we paid less attention to the changes in cytological behaviour caused by TIMP‐2, such as cell cycle changes, cell crawling and penetration ability, cell pyroptosis, ferroptosis, etc. The dramatic changes in cytological behaviour are also beneficial in our search for opportunities to treat 5‐Fu‐resistant CRC. In terms of animal experiments, if the state of CRC tumour resistance to 5‐Fu can be simulated in a mouse model, the changes in the expression level of TIMP‐2, the expression intensity of the JAK–STAT signalling pathway, and the effect of target treating the 5‐Fu resistance could be further studied. This will serve as strong evidence for our experimental conclusions. The cell and animal experiments mentioned above are the next steps of our research group.

Here, we also used a small molecule inhibitor targeting the JAK–STAT signalling pathway to verify whether inhibiting the JAK–STAT signalling pathway could reverse the 5‐Fu resistance of CRC cells [48]. The experimental results showed that LY2784544 effectively overcomes the 5‐Fu resistance of CRC. Although more recombinant TIMP‐2 was added to the culture medium of cell lines, the small molecule inhibitor LY2784544 could still act on the downstream JAK–STAT signalling pathway to sensitise CRC cells to 5‐Fu. How LY2784544 affects the biological behaviour of CRC cells and whether it can play an equally significant role in animal models requires our follow‐up experimental studies to demonstrate.

As for why the inhibitor LY2784544 can synergistically enhance the cytotoxic effects of 5‐FU, our experimental results partially demonstrate that JAK2 can prevent STAT activation and the transcription of genes that promote cell survival and proliferation. This action complements the disruption of DNA and RNA synthesis caused by 5‐FU. Additionally, we speculate that the combination may modulate the tumour microenvironment, as the role of the JAK–STAT pathway in immune regulation suggests that its inhibition could activate immune responses against cancer cells or reduce immunosuppressive factors. Furthermore, LY2784544 may counteract resistance mechanisms that can develop in response to 5‐FU, such as changes in drug metabolism or transport. These mechanisms collectively contribute to the synergistic therapeutic effect of LY2784544 and 5‐FU on sensitive cancer cells. Targeted small‐molecule inhibitors can be studied in clinical trials after reliable laboratory evaluation, including animal experiments. Of course, the dose of LY2784544 needs to be carefully evaluated by researchers. After all, it will also have some bad clinical side effects. Nevertheless, 5‐Fu combined with LY2784544 is a very promising way to treat CRC patients, which may change the state of 5‐Fu resistance during their prolonged treatment.

## 5 Conclusions

In conclusion, we found that elevated TIMP‐2 expression levels correlated with the 5‐Fu resistance status of CRC cells, both at the cellular and the patient serum level. Cytological experiments confirmed that TIMP‐2 is a key cytokine mediating CRC resistance to 5‐Fu. Further, we showed that TIMP‐2 causes resistance to 5‐Fu drugs by constitutively activating JAK–STAT. Since the small molecule inhibitor LY2784544 targeting the JAK–STAT signalling pathway can effectively reverse the resistance of CRC to 5‐Fu, it is a new research target for treating this intractable disease.

## Author Contributions

**Chuchu Xu:** funding acquisition (equal), methodology (equal), resources (equal), writing – original draft (equal). **Renjun Zhu:** methodology (equal), resources (equal). **Qingfeng Dai:** formal analysis (equal), software (equal). **Yaoqing Li:** data curation (equal), funding acquisition (equal), methodology (equal), resources (equal). **Gengyuan Hu:** methodology (equal), resources (equal). **Kelong Tao:** funding acquisition (equal), methodology (equal), resources (equal). **Yuhong Xu:** funding acquisition (equal), methodology (equal), resources (equal). **Guangen Xu:** conceptualization (equal), investigation (equal), supervision (equal). **Guolin Zhang:** conceptualization (equal), investigation (equal funding), writing – review and editing (lead).

## Ethics Statement

The human and animal ethics involved in this experiment were approved by the ethical approval agency at Shaoxing People's Hospital (Shaoxing Hospital, Zhejiang University School of Medicine). Study number: 2021‐K‐Y‐158‐01.

## Consent

The authors affirm that human research participants provided informed consent for publication.

## Conflicts of Interest

The authors declare no conflicts of interest.

## Data Availability

Data supporting the findings of this study are available from the Department of Gastrointestinal Surgery, Shaoxing People's Hospital, but the availability of these data is limited, they are used under the licence of the current study and are therefore not available publicly available. Data are available from the authors upon reasonable request and with permission of the Department of Gastrointestinal Surgery, Shaoxing People's Hospital.

## References

[1] H. Sung , J. Ferlay , R. L. Siegel , et al., “Global Cancer Statistics 2020: GLOBOCAN Estimates of Incidence and Mortality Worldwide for 36 Cancers in 185 Countries,” CA: a Cancer Journal for Clinicians 71 (2021): 209–249, 10.3322/caac.21660. 33538338

[2] L. H. Biller and D. Schrag , “Diagnosis and Treatment of Metastatic Colorectal Cancer: A Review,” JAMA 325 (2021): 669–685, 10.1001/jama.2021.0106. 33591350

[3] R. L. Siegel , K. D. Miller , H. E. Fuchs , and A. Jemal , “Cancer Statistics, 2021,” CA: A Cancer Journal for Clinicians 71 (2021): 7–33, 10.3322/caac.21654. 33433946

[4] E. Dekker , P. J. Tanis , J. L. A. Vleugels , P. M. Kasi , and M. B. Wallace , “Colorectal Cancer,” Lancet 394 (2019): 1467–1480, 10.1016/S0140-6736(19)32319-0. 31631858

[5] D. B. Longley , D. P. Harkin , and P. G. Johnston , “5‐Fluorouracil: Mechanisms of Action and Clinical Strategies,” Nature Reviews. Cancer 3 (2003): 330–338, 10.1038/nrc1074. 12724731

[6] C. Sethy and C. N. Kundu , “5‐Fluorouracil (5‐FU) Resistance and the New Strategy to Enhance the Sensitivity Against Cancer: Implication of DNA Repair Inhibition,” Biomedicine & Pharmacotherapy 137 (2021): 111285, 10.1016/j.biopha.2021.111285. 33485118

[7] S. Blondy , V. David , M. Verdier , M. Mathonnet , A. Perraud , and N. Christou , “5‐Fluorouracil Resistance Mechanisms in Colorectal Cancer: From Classical Pathways to Promising Processes,” Cancer Science 111 (2020): 3142–3154, 10.1111/cas.14532. 32536012 PMC7469786

[8] Y. Shen , M. Tong , Q. Liang , et al., “Epigenomics Alternations and Dynamic Transcriptional Changes in Responses to 5‐Fluorouracil Stimulation Reveal Mechanisms of Acquired Drug Resistance of Colorectal Cancer Cells,” Pharmacogenomics Journal 18 (2018): 23–28, 10.1038/tpj.2016.91. 28045128 PMC5817391

[9] F. D. Pouya , M. Gazouli , Y. Rasmi , D. I. Lampropoulou , and M. Nemati , “MicroRNAs and Drug Resistance in Colorectal Cancer With Special Focus on 5‐Fluorouracil,” Molecular Biology Reports 49 (2022): 5165–5178, 10.1007/s11033-022-07227-1. 35212928

[10] S. Vodenkova , T. Buchler , K. Cervena , V. Veskrnova , P. Vodicka , and V. Vymetalkova , “5‐Fluorouracil and Other Fluoropyrimidines in Colorectal Cancer: Past, Present and Future,” Pharmacology & Therapeutics 206 (2020): 107447, 10.1016/j.pharmthera.2019.107447. 31756363

[11] L. L. Lu , X. H. Chen , G. Zhang , et al., “CCL21 Facilitates Chemoresistance and Cancer Stem Cell‐Like Properties of Colorectal Cancer Cells Through AKT/GSK‐3β/Snail Signals,” Oxidative Medicine and Cellular Longevity 2016 (2016): 5874127, 10.1155/2016/5874127. 27057280 PMC4707330

[12] D. Dabkeviciene , V. Jonusiene , V. Zitkute , et al., “The Role of Interleukin‐8 (CXCL8) and CXCR2 in Acquired Chemoresistance of Human Colorectal Carcinoma Cells HCT116,” Medical Oncology 32 (2015): 258, 10.1007/s12032-015-0703-y. 26519257

[13] X. Mao , J. Xu , W. Wang , et al., “Crosstalk Between Cancer‐Associated Fibroblasts and Immune Cells in the Tumor Microenvironment: New Findings and Future Perspectives,” Molecular Cancer 20 (2021): 131, 10.1186/s12943-021-01428-1. 34635121 PMC8504100

[14] M. T. Chow and A. D. Luster , “Chemokines in Cancer,” Cancer Immunology Research 2 (2014): 1125–1131, 10.1158/2326-6066.cir-14-0160. 25480554 PMC4258879

[15] V. S. Jones , R. Y. Huang , L. P. Chen , Z. S. Chen , L. Fu , and R. P. Huang , “Cytokines in Cancer Drug Resistance: Cues to New Therapeutic Strategies,” Biochimica et Biophysica Acta 1865 (2016): 255–265, 10.1016/j.bbcan.2016.03.005. 26993403

[16] R. A. Wagenaar‐Miller , L. Gorden , and L. M. Matrisian , “Matrix Metalloproteinases in Colorectal Cancer: Is It Worth Talking About?,” Cancer Metastasis Reviews 23 (2004): 119–135. 15000153 10.1023/a:1025819214508

[17] A. Winer , S. Adams , and P. Mignatti , “Matrix Metalloproteinase Inhibitors in Cancer Therapy: Turning Past Failures Into Future Successes,” Molecular Cancer Therapeutics 17 (2018): 1147–1155, 10.1158/1535-7163.MCT-17-0646. 29735645 PMC5984693

[18] L. Herszényi , I. Hritz , G. Lakatos , M. Z. Varga , and Z. Tulassay , “The Behavior of Matrix Metalloproteinases and Their Inhibitors in Colorectal Cancer,” International Journal of Molecular Sciences 13 (2012): 13240–13263, 10.3390/ijms131013240. 23202950 PMC3497324

[19] G. A. Cabral‐Pacheco , I. Garza‐Veloz , C. Castruita‐de la Rosa , et al., “The Roles of Matrix Metalloproteinases and Their Inhibitors in Human Diseases,” International Journal of Molecular Sciences 21 (2020): 9739, 10.3390/ijms21249739. 33419373 PMC7767220

[20] H. W. Jackson , V. Defamie , P. Waterhouse , and R. Khokha , “TIMPs: versatile extracellular regulators in cancer,” Nature Reviews. Cancer 17 (2017): 38–53, 10.1038/nrc.2016.115. 27932800

[21] D. Peeney , S. M. Jensen , N. P. Castro , et al., “TIMP‐2 Suppresses Tumor Growth and Metastasis in Murine Model of Triple‐Negative Breast Cancer,” Carcinogenesis 41 (2020): 313–325, 10.1093/carcin/bgz172. 31621840 PMC7221506

[22] A. Chowdhury , R. Brinson , B. Wei , and W. G. Stetler‐Stevenson , “Tissue Inhibitor of Metalloprotease‐2 (TIMP‐2): Bioprocess Development, Physicochemical, Biochemical, and Biological Characterization of Highly Expressed Recombinant Protein,” Biochemistry 56 (2017): 6423–6433, 10.1021/acs.biochem.7b00700. 29140689 PMC6322544

[23] M. Łukaszewicz‐Zając , B. Mroczko , K. Guzińska‐Ustymowicz , et al., “Matrix Metalloproteinase 2 (MMP‐2) and Their Tissue Inhibitor 2 (TIMP‐2) in Gastric Cancer Patients,” Advances in Medical Sciences 58 (2013): 235–243, 10.2478/ams-2013-0018. 24384769

[24] L. Barabás , I. Hritz , G. István , Z. Tulassay , and L. Herszényi , “The Behavior of MMP‐2, MMP‐7, MMP‐9, and Their Inhibitors TIMP‐1 and TIMP‐2 in Adenoma‐Colorectal Cancer Sequence,” Digestive Diseases 39 (2021): 217–224, 10.1159/000511765. 32961536

[25] J. M. Azevedo Martins and S. H. Rabelo‐Santos , “Tumoral and Stromal Expression of MMP‐2, MMP‐9, MMP‐14, TIMP‐1, TIMP‐2, and VEGF‐A in Cervical Cancer Patient Survival: A Competing Risk Analysis,” BMC Cancer 20 (2020): 660, 10.1186/s12885-020-07150-3. 32669083 PMC7364527

[26] L. Zhu , H. Yu , S. Y. Liu , et al., “Prognostic Value of Tissue Inhibitor of Metalloproteinase‐2 Expression in Patients With Non‐Small Cell Lung Cancer: A Systematic Review and Meta‐Analysis,” PLoS One 10 (2015): e0124230, 10.1371/journal.pone.0124230. 25905787 PMC4408055

[27] W. Wang , D. Li , L. Xiang , et al., “TIMP‐2 Inhibits Metastasis and Predicts Prognosis of Colorectal Cancer via Regulating MMP‐9,” Cell Adhesion & Migration 13 (2019): 273–284, 10.1080/19336918.2019.1639303. 31293204 PMC6629184

[28] R. M. Escalona , M. Bilandzic , P. Western , et al., “TIMP‐2 Regulates Proliferation, Invasion and STAT3‐Mediated Cancer Stem Cell‐Dependent Chemoresistance in Ovarian Cancer Cells,” BMC Cancer 20 (2020): 960, 10.1186/s12885-020-07274-6. 33023532 PMC7542139

[29] X. Chen , S. L. Zhong , P. Lu , et al., “miR‐4443 Participates in the Malignancy of Breast Cancer,” PLoS One 11 (2016): e0160780, 10.1371/journal.pone.0160780. 27504971 PMC4978484

[30] F. Tavakoli , R. Jahanban‐Esfahlan , K. Seidi , et al., “Effects of Nano‐Encapsulated Curcumin‐Chrysin on Telomerase, MMPs and TIMPs Gene Expression in Mouse B16F10 Melanoma Tumour Model,” Artificial Cells, Nanomedicine, and Biotechnology 46 (2018): 75–86, 10.1080/21691401.2018.1452021. 29607740

[31] C. Liongue , R. Sertori , and A. C. Ward , “Evolution of Cytokine Receptor Signaling,” Journal of Immunology 197 (2016): 11–18, 10.4049/jimmunol.1600372. 27317733

[32] R. Morris , N. J. Kershaw , and J. J. Babon , “The Molecular Details of Cytokine Signaling via the JAK/STAT Pathway,” Protein Science 27 (2018): 1984–2009, 10.1002/pro.3519. 30267440 PMC6237706

[33] P. Xin , X. Xu , C. Deng , et al., “The Role of JAK/STAT Signaling Pathway and Its Inhibitors in Diseases,” International Immunopharmacology 80 (2020): 106210, 10.1016/j.intimp.2020.106210. 31972425

[34] K. L. Owen , N. K. Brockwell , and B. S. Parker , “JAK‐STAT Signaling: A Double‐Edged Sword of Immune Regulation and Cancer Progression,” Cancers (Basel) 11 (2019): 2002, 10.3390/cancers11122002. 31842362 PMC6966445

[35] Z. Yan , S. A. Gibson , J. A. Buckley , H. Qin , and E. N. Benveniste , “Role of the JAK/STAT Signaling Pathway in Regulation of Innate Immunity in Neuroinflammatory Diseases,” Clinical Immunology 189 (2018): 4–13, 10.1016/j.clim.2016.09.014. 27713030 PMC5573639

[36] S. Grivennikov , E. Karin , J. Terzic , et al., “IL‐6 and Stat3 Are Required for Survival of Intestinal Epithelial Cells and Development of Colitis‐Associated Cancer,” Cancer Cell 15 (2009): 103–113, 10.1016/j.ccr.2009.01.001. 19185845 PMC2667107

[37] G. Huang , X. Wu , S. Li , X. Xu , H. Zhu , and X. Chen , “The Long Noncoding RNA CASC2 Functions as a Competing Endogenous RNA by Sponging miR‐18a in Colorectal Cancer,” Scientific Reports 6 (2016): 26524, 10.1038/srep26524. 27198161 PMC4873821

[38] A. N. Gargalionis , K. A. Papavassiliou , and A. G. Papavassiliou , “Targeting STAT3 Signaling Pathway in Colorectal Cancer,” Biomedicine 9 (2021): 1016, 10.3390/biomedicines9081016. PMC8392110 34440220

[39] S. J. Thomas , J. A. Snowden , M. P. Zeidler , and S. J. Danson , “The Role of JAK/STAT Signalling in the Pathogenesis, Prognosis and Treatment of Solid Tumours,” British Journal of Cancer 113 (2015): 365–371, 10.1038/bjc.2015.233. 26151455 PMC4522639

[40] K. Meissl , S. Macho‐Maschler , M. Müller , and B. Strobl , “The Good and the Bad Faces of STAT1 in Solid Tumours,” Cytokine 89 (2017): 12–20, 10.1016/j.cyto.2015.11.011. 26631912

[41] N. N. Khodarev , B. Roizman , and R. R. Weichselbaum , “Molecular Pathways: Interferon/stat1 Pathway: Role in the Tumor Resistance to Genotoxic Stress and Aggressive Growth,” Clinical Cancer Research 18 (2012): 3015–3021, 10.1158/1078-0432.ccr-11-3225. 22615451

[42] C. Güngör , H. Zander , K. E. Effenberger , et al., “Notch Signaling Activated by Replication Stress‐Induced Expression of Midkine Drives Epithelial‐Mesenchymal Transition and Chemoresistance in Pancreatic Cancer,” Cancer Research 71 (2011): 5009–5019, 10.1158/0008-5472.can-11-0036. 21632553

[43] Y. Lu , B. Yan , H. Guo , et al., “Effect of Midkine on Gemcitabine Resistance in Biliary Tract Cancer,” International Journal of Molecular Medicine 41 (2018): 2003–2011, 10.3892/ijmm.2018.3399. 29344648 PMC5810218

[44] G. Soslau , C. Mason , S. Lynch , et al., “Intracellular Matrix Metalloproteinase‐2 (MMP‐2) Regulates Human Platelet Activation via Hydrolysis of Talin,” Thrombosis and Haemostasis 111 (2014): 140–153, 10.1160/th13-03-0248. 24136115

[45] L. Wei , J. Sun , N. Zhang , et al., “Noncoding RNAs in Gastric Cancer: Implications for Drug Resistance,” Molecular Cancer 19 (2020): 62, 10.1186/s12943-020-01185-7. 32192494 PMC7081551

[46] B. Du and J. S. Shim , “Targeting Epithelial‐Mesenchymal Transition (EMT) to Overcome Drug Resistance in Cancer,” Molecules 21 (2016): 965, 10.3390/molecules21070965. 27455225 PMC6273543

[47] G. Zhang , X. Luo , Z. Wang , et al., “TIMP‐2 Regulates 5‐Fu Resistance via the ERK/MAPK Signaling Pathway in Colorectal Cancer,” Aging 14 (2022): 297–315, 10.18632/aging.203793. 35022331 PMC8791226

[48] W. Zhou , W. Sun , M. M. H. Yung , et al., “Autocrine Activation of JAK2 by IL‐11 Promotes Platinum Drug Resistance,” Oncogene 37 (2018): 3981–3997, 10.1038/s41388-018-0238-8. 29662190 PMC6054535

